# Chitosan-based nanoparticles for improved anticancer efficacy and bioavailability of mifepristone

**DOI:** 10.3762/bjnano.7.178

**Published:** 2016-11-28

**Authors:** Huijuan Zhang, Fuqiang Wu, Yazhen Li, Xiping Yang, Jiamei Huang, Tingting Lv, Yingying Zhang, Jianzhong Chen, Haijun Chen, Yu Gao, Guannan Liu, Lee Jia

**Affiliations:** 1 College of Chemistry, Fuzhou University, Fuzhou 350108, China; 2 School of Pharmacy, Fujian University of Traditional Chinese Medicine, Fuzhou 350108, China,; 3 College of Life Sciences, China Jiliang University, Hangzhou, Zhejiang, 310018, China

**Keywords:** anticancer, chitosan, drug delivery, mifepristone, nanoparticles, pharmacokinetics, sustained release

## Abstract

In addition to its well-known abortifacient effect, mifepristone (MIF) has been used as an anticancer drug for various cancers in many studies with an in-depth understanding of the mechanism of action. However, application of MIF is limited by its poor water solubility and low oral bioavailability. In this work, we developed a drug delivery system based on chitosan nanoparticles (CNs) to improve its bioavailability and anticancer activity. The MIF-loaded chitosan nanoparticles (MCNs) were prepared by convenient ionic gelation techniques between chitosan (Cs) and tripolyphosphate (TPP). The preparation conditions, including Cs concentration, TPP concentration, Cs/MIF mass ratio, and pH value of the TPP solution, were optimized to gain better encapsulation efficiency (EE) and drug loading capacity (DL). MCNs prepared with the optimum conditions resulted in spherical particles with an average size of 200 nm. FTIR and XRD spectra verified that MIF was successfully encapsulated in CNs. The EE and DL of MCNs determined by HPLC were 86.6% and 43.3%, respectively. The in vitro release kinetics demonstrated that MIF was released from CNs in a sustained-release manner. Compared with free MIF, MCNs demonstrated increased anticancer activity in several cancer cell lines. Pharmacokinetic studies in male rats that were orally administered MCNs showed a 3.2-fold increase in the area under the curve from 0 to 24 h compared with free MIF. These results demonstrated that MCNs could be developed as a potential delivery system for MIF to improve its anticancer activity and bioavailability.

## Introduction

Mifepristone (RU486, MIF) acts as a progesterone receptor (PR) modulator and has been widely used for emergency contraception and to provoke early-stage abortion [1–2]. Recently, it has been approved by the Federal Drug Administration (FDA) to treat hyperglycemia associated with Cushing’s syndrome [3]. Besides its antiglucocorticoid and antiprogestogen activity, MIF has been shown to promote anticancer activity in cancer cell lines and in clinical trials [4–5]. However, some side effects of MIF including nausea, vomiting, and bleeding are still observed in the clinic trails [6]. Because the side effects of MIF are closely related with its dosage, some studies have been implemented to find the optimum dosage of MIF [7] or to develop new formulations for MIF to improve its bioavailability [8].

Chitosan (Cs) is a basic polysaccharide found in nature with good biocompatibility and biodegradability [9]. It possesses various bioactivities such as anti-inflammatory, antibacterial, antifungal, muco-adhesive, and antitumor effects [10–11]. Therefore, chitosan has been widely used as a biomaterial or adjuvant in disease therapy [12], tissue engineering, and drug delivery [13]. Owning to the reactive amino side groups, chitosan could be made available via chemical modifications or ionic interactions [14]. Chitosan-bearing protonated amino groups could interact with a wide variety of natural or synthetic anionic species, such as negatively charged proteins, DNA [15–19], and some synthetic basic polymers such as sodium tripolyphosphate (TPP) [20–21] to form ionic complexes. This ionic gelation method to prepare Cs/TPP nanoparticles (CNs) with the advantages of simple operation, low equipment requirements, low cost, good repeatability, environmentally friendly, and easy large-scale preparation, has been extensively studied for obtaining nanocarrier systems with a good capacity of drug encapsulation and an adjustable drug release rate [22–23].

The aim of this work was to prepare MIF-encapsulated CNs (MCNs) to regulate the drug release rate of MIF for bioavailability improvement, and meanwhile, enhance the antitumor effect of MIF by the auxiliary anticancer functionality of Cs. The ionic gelation technique was used to prepare MCNs. The preparation conditions for MCNs were optimized and the physiochemical properties of MCNs were characterized. Then, the in vitro drug release behavior of MCNs was determined. Finally, the anticancer activity of MCNs was studied in several cancer cell lines and the pharmacokinetic studies of MCNs were performed in male rats.

## Results and Discussion

### Preparation and optimization of MCNs

**Figure 1:**
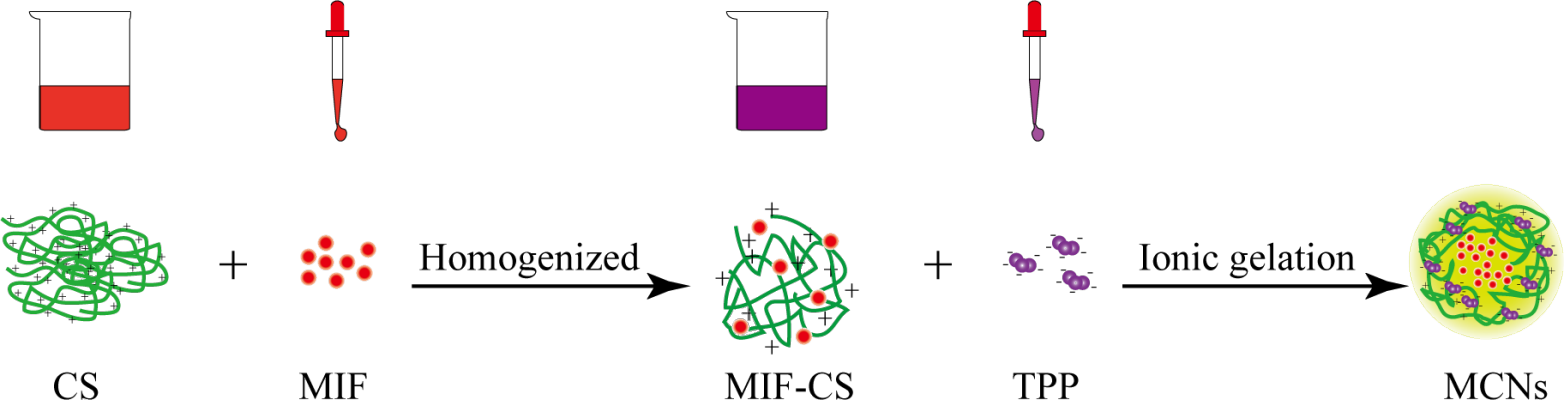
Schematic illustration of the preparation procedure for MCNs. MIF-loaded CNs were prepared by a convenient ionic gelation technique. Cs was firstly mixed with MIF to afford a homogeneous solution then interacted with a TPP solution to allow MIF to be encapsulated during the ionic gelation process.

In this study, MIF-loaded CNs were prepared by a convenient ionic gelation technique (Figure 1). This technique has been widely used to prepare CNs as drug delivery systems for a variety of drugs, including either hydrophobic drugs or hydrophilic protein drugs [22,24]. Because of the strong hydrophobicity of MIF, it is hard to load MIF into blank CNs after the ionic gelation process. Therefore, Cs was firstly mixed with MIF to afford a homogeneous solution. This solution was then interacted with a TPP solution to allow MIF to be encapsulated during the ionic gelation process. Because Cs was insoluble in water, 2% aqueous acetic acid solution was used as a solvent for Cs [25]. Ethanol and Tween-80 (1:1 v/v) were used as the organic solvent and detergent, respectively, for MIF because of their low toxic potential and emulsifying activity for dissolution of the hydrophobic components. In the preparation process, it was reported that several factors such as Cs concentration, TPP concentration, Cs/TPP mass ratio could have an influence on particle size, encapsulation efficiency (EE), and drug loading capacity (DL) of the nanoparticles [23,26]. In order to select optimum conditions for preparation of MCNs, the effects of Cs concentration, TPP concentration, and Cs/MIF mass ratio on the EE and DL of nanoparticles were investigated [25]. Because the pH values have great influence on the properties of MIF [27], the pH value of the TPP solution was also optimized.

**Figure 2:**
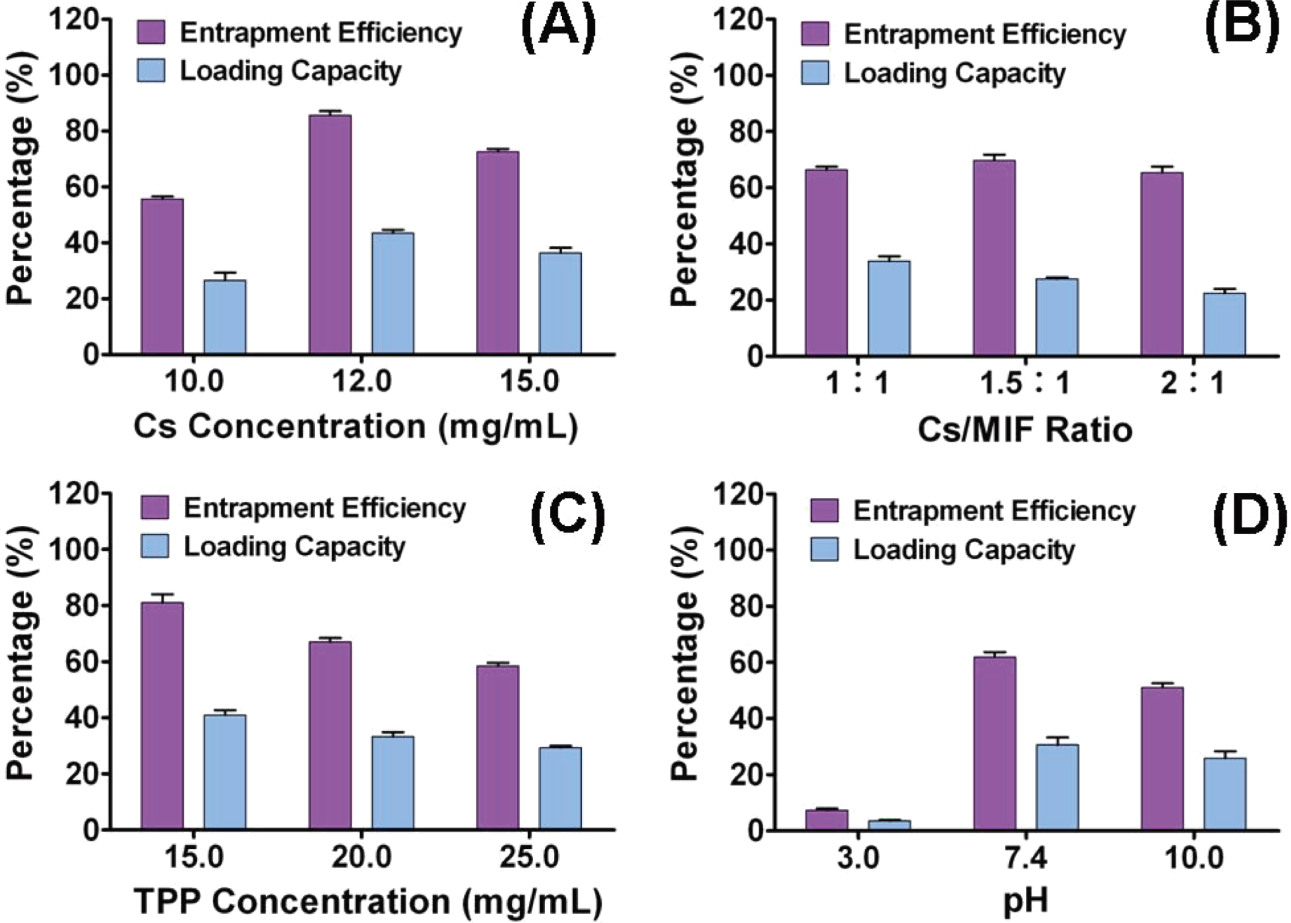
The influence of the preparation conditions including Cs concentration (A), Cs/MIF mass ratio (B), TPP concentration (C), and pH of TPP solution (D) on the encapsulation efficiency (EE) and drug loading capacity (DL) of MCNs. The optimum preparation conditions are: Cs concentration of 12 mg/mL, Cs/MIF mass ratio of 1:1, TPP concentration of 15 mg/mL, and TPP solution pH 7. When a parameter is changed, the other variables are selected with the optimum conditions.

As shown in Figure 2, the EEs or DLs of different nanoparticle formulations were varied with the change of Cs concentration, TPP concentration, and Cs/MIF mass ratio, and the pH value of TPP solution. Firstly, we kept the Cs/MIF ratio as 1:1, TPP concentration as 15 mg/mL, and the pH value of the TPP solution as 7, and investigated the effects of Cs concentration on EE and DL of MCNs. The results showed that a Cs concentration of 12 mg/mL resulted in higher EE and DL. Cs with low concentration could not entirely entrap MIF, therefore the EE and DL were lower. However, the viscosity of the Cs solution increases with increasing concentration, and the EE will decrease as the degree of dispersion of Cs decreases. In the same way, we found the TPP concentration also has a great effect on EE and DL of MCNs. With the increase of the TPP concentration, the EE and DL of MCNs dropped significantly. This could be due to the increased reaction degree between Cs and TPP, leading to cross-linking of nanoparticles with abnormal morphology, which resulted in decreased EE and DL. The pH value of the TPP solution is the most important factor affecting EE and DL of MCNs. The MCNs presented the best EE and DL with a pH 7. This implied better stability and solubility of MIF in neutral pH conditions. The optimum preparation conditions are: Cs concentration of 12 mg/mL, Cs/MIF ratio of 1:1, TPP concentration of 15 mg/mL, and pH value of TPP solution of 7. MCNs prepared with the optimum conditions were then subjected to the following studies.

### Characterization

#### Fourier transform infrared (FTIR) analysis

**Figure 3:**
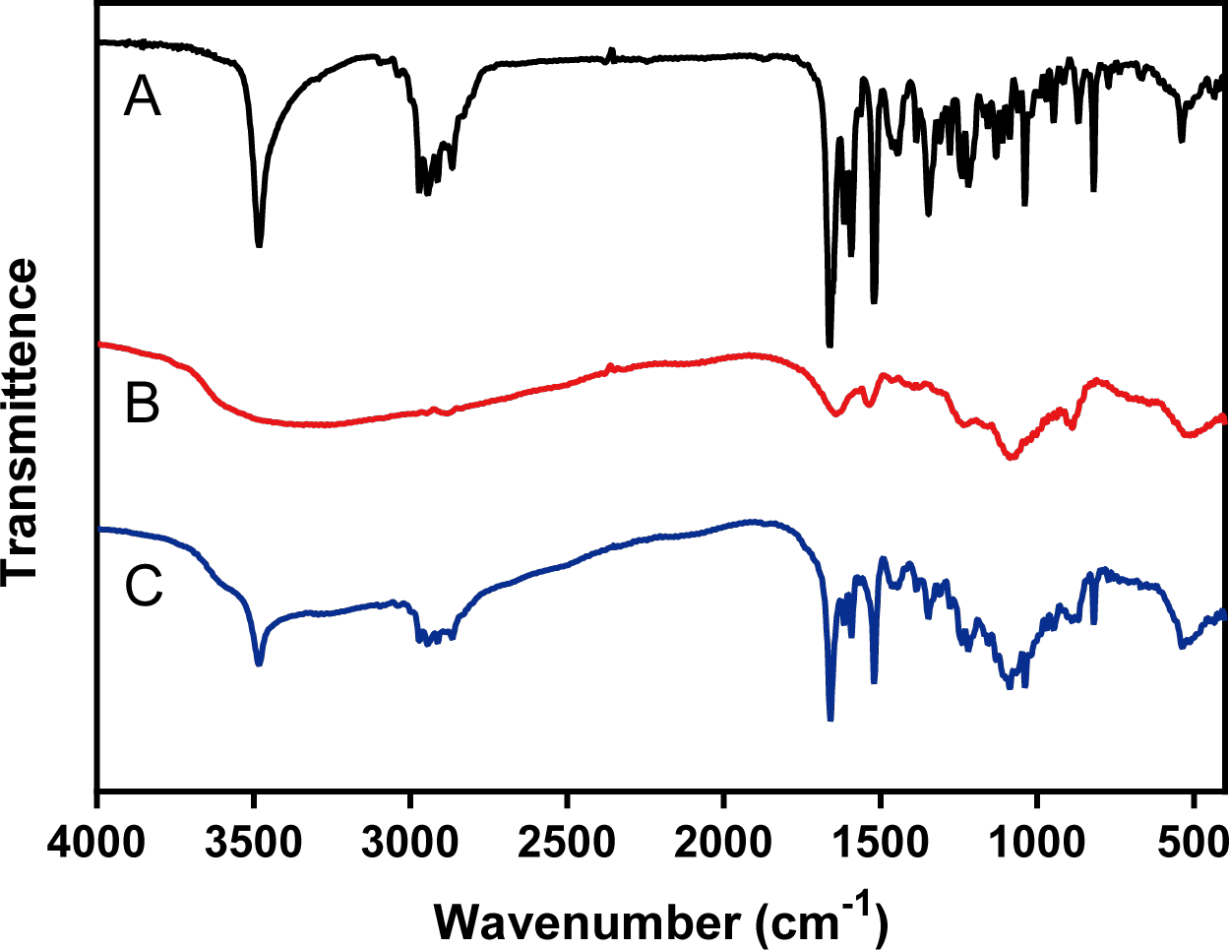
FITR spectra of (A) MIF, (B) blank CNs, and (C) MCNs. The MCNs formulation showed the characteristic absorption peaks of both MIF and CNs.

Figure 3 shows the FTIR spectra of the MIF, blank CNs, and the MCNs. There were five characteristic peaks of MIF including a band at 3481cm^−1^ due to –OH stretching vibrations of the hydroxyl group, a broad band between 2941 and 2866 cm^−1^ corresponding to the saturated C–H stretching vibrations of the various methyl and methylene groups, and two other sharp absorption bands at 1657 cm^−1^ and 1517 cm^−1^ corresponding to the C–H stretching vibrations of aromatic nucleus. In the FTIR spectrum of blank CNs, characteristic peaks were displayed at 1514 cm^−1^ due to +NH_3_ groups [28], and other peaks located at 1086 cm^−1^ due to P–O stretching vibration. The MCNs formulation showed the characteristic absorption peaks of both MIF and CNs, which proved that MIF was successfully wrapped in Cs nanoparticles.

#### X-ray diffraction (XRD) analysis

**Figure 4:**
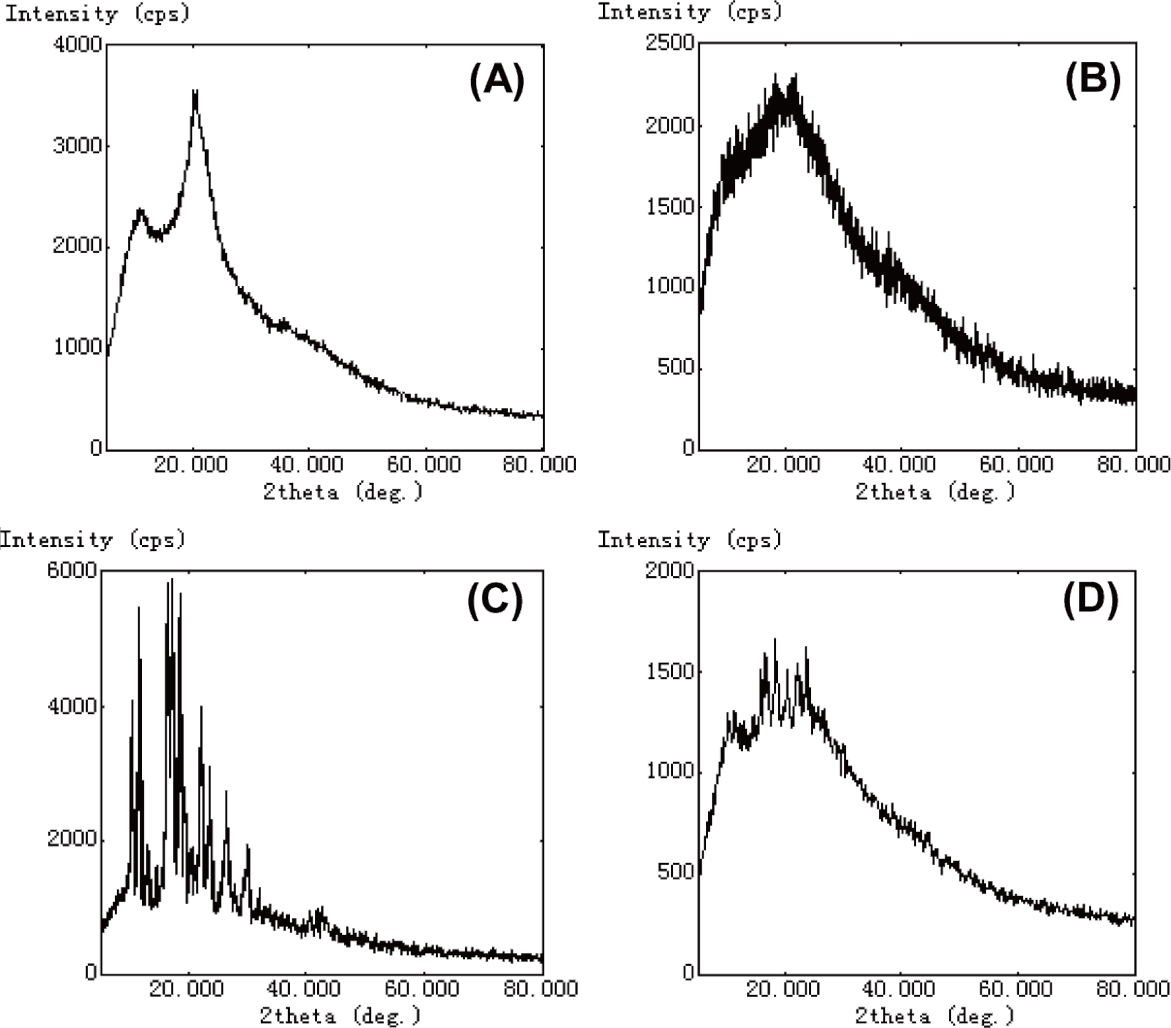
XRD spectra of (A) Cs, (B) blank CNs, (C) MIF, and (D) MCNs. The XRD analysis of MCNs showed the detailed peaks of MIF superimposed over the amorphous features of CNs.

The XRD patterns were studied to observe the change in crystallinity of the MIF in the carrier system of CNs. Figure 4 shows the XRD pattern of the Cs, blank CNs, MIF, and MCNs. In the XRD patterns of Cs (Figure 4A), two intense diffraction peaks of crystallinity were observed. A broadening peak with lower intensity at about 2θ between 10° and 30° was observed in Figure 4B as compared with Figure 4A, indicating the decrease of the crystallinity of Cs structure and the presence of Cs in amorphous forms in the nanoparticles [6]. Figure 4C shows the XRD pattern of MIF, which displayed a distinct spectrum. The XRD analysis of MCNs showed the detailed peaks of MIF superimposed over the broad, amorphous structure of CNs (Figure 4D), indicating that the MIF was embedded in the CNs.

#### Size and surface morphology of MCNs

**Figure 5:**
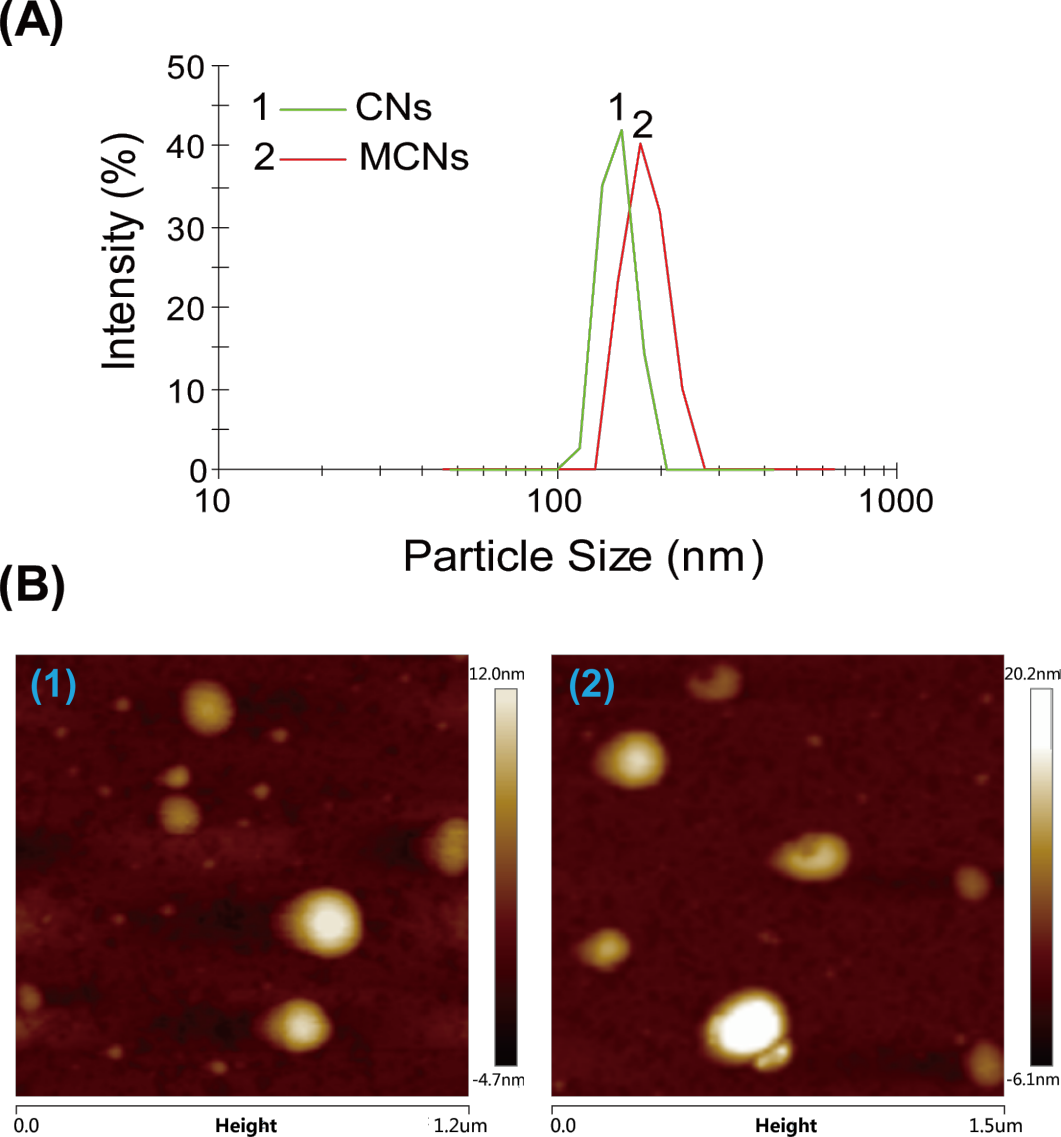
(A) DLS measurements of (1) CNs and (2) MCNs. (B) AFM images of (1) CNs and (2) MCNs.

The diameters of CNs and MCNs measured by a Malvern particle size analyzer were found to be from 180 to 200 nm (Figure 5A). The morphologies of CNs and MCNs were observed using AFM (Figure 5B). It could be seen that the nanoparticles were spherical in shape with a relatively smooth surface. The tiny dots in the AFM images might be due to the free chitosan. No obvious morphological changes between CNs and MCNs could be found. The results showed that the horizontal distance of MCNs increased from about 150 nm to 200 nm and the vertical distance of MCNs increased from about 20 nm to 30 nm after MIF entrapment. It was reported that the concentration of TPP and the concentration of Cs have effects on the size of chitosan nanoparticles [26,29]. However, considering that the gastrointestinal absorption and bioavailability of nanoparticles designed for oral administration was closely related to the properties of the composition and the ingredient for specific targeting [30], the influencing factors of the size of MCNs were not studied in this work.

### In vitro release study

**Figure 6:**
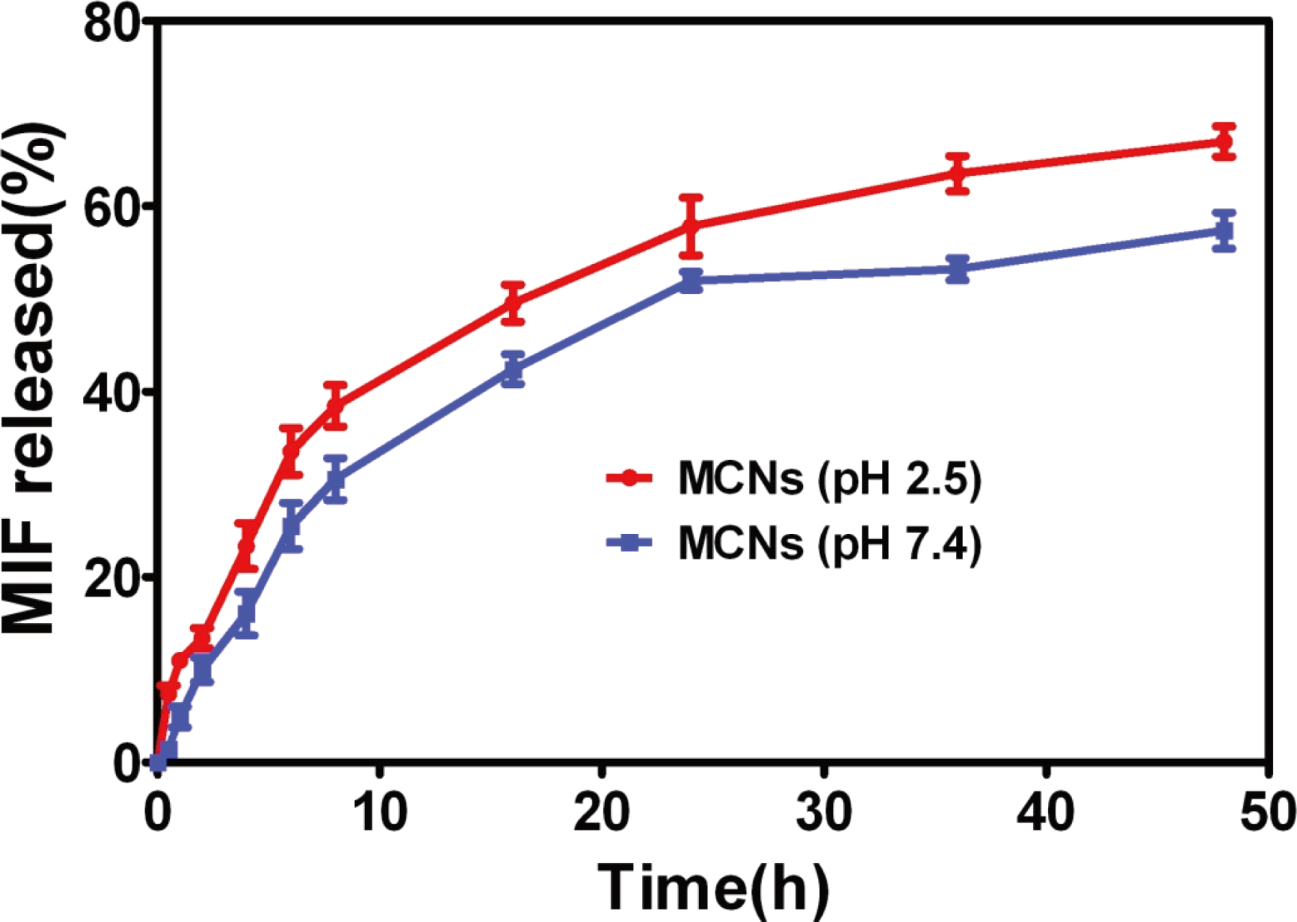
In vitro release profiles of MIF from MCNs at pH 2.5 and 7.4. The release of MIF from the dialysis tube containing MCNs immersed in 0.1 M PBS (pH 7.4) or 0.1 M PBS (pH 2.5) containing 1% of ethanol.

The in vitro release study of MIF from MCNs was performed in PBS buffer solutions at pH 7.4 or pH 2.5 to simulate the different pH conditions of the gastrointestinal system [31–32]. To enhance the solubility of MIF, 1% of ethanol was added to the incubation medium. The samples were taken out of the medium at designed time points and the released MIF was quantified by HPLC. The release rate of MIF from MCNs showed a sustained release profile in both buffers, and the release rate of MIF from MCNs was very fast at pH 2.5 (Figure 6). This is because MIF, with weakly basic nitrogen, is more likely to dissolve in acidic solution [33]. The sustained-release manner of MCNs could prolong the time of drug absorption in the gastrointestinal tract, which might be beneficial to enhanced bioavailability of MIF [31,34]. The sustained-release phenomenon also proved that TPP is an appropriate crosslinking agent for controlled drug release of CNs.

### In vitro anticancer effects

**Figure 7:**
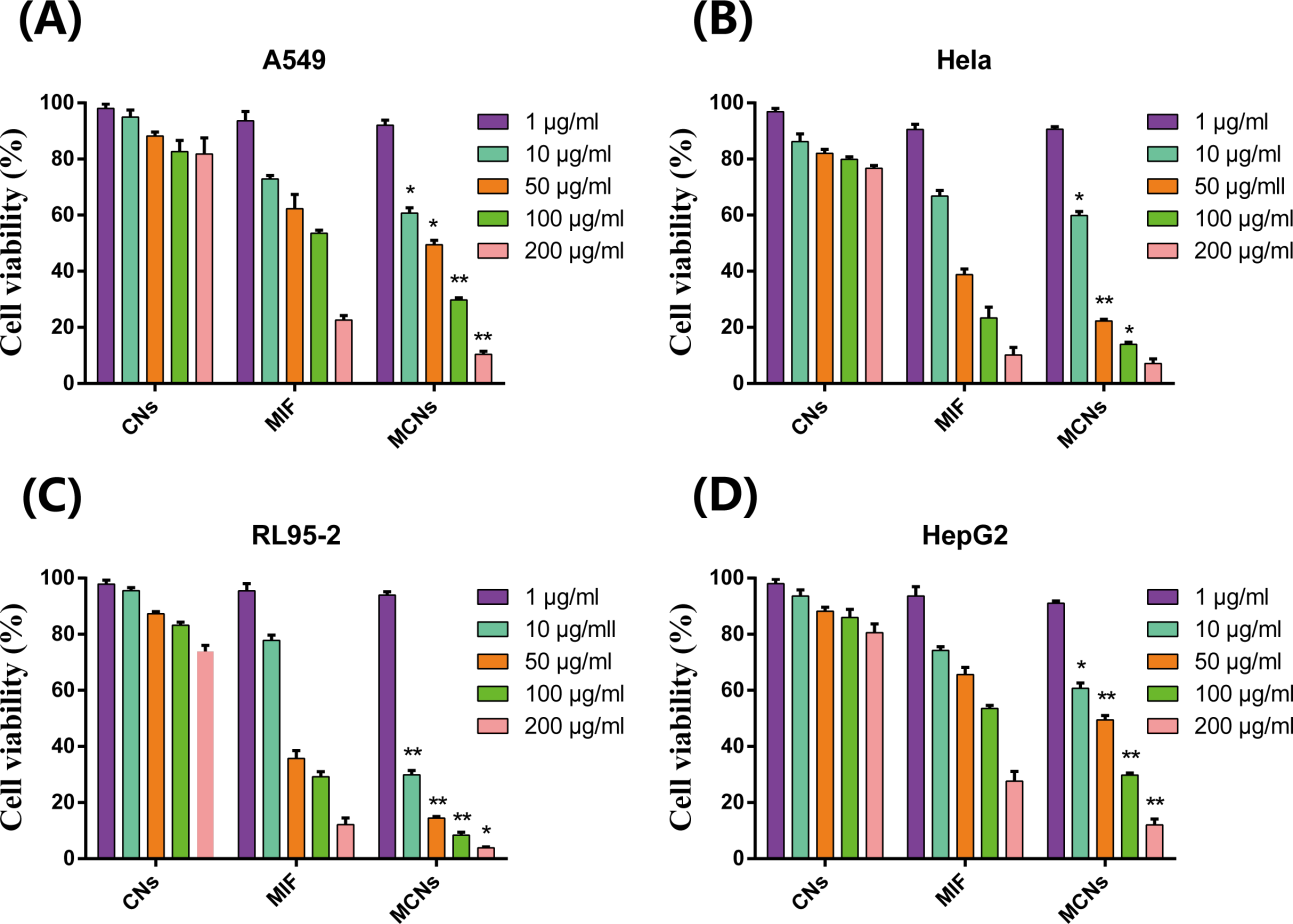
In vitro cytotoxicity of CNs, MIF and MCNs against A549 (A), Hela (B), RL95-2 (C), and HepG2 cells (D). Cells were incubated with different concentrations (1, 10, 50, 100, 200 μg/mL) of blank CNs, free MIF, or MCNs for 48 h at 37 °C before subjecting them to MTT assay. *p < 0.05 and **p < 0.01 compared with the MIF group by the Student’s *t*-test.

The cytotoxicity of the MCNs was tested in four different cancer cell lines A549 (human lung adenocarcinoma), Hela (human cervical epithelioid carcinoma), RL95-2 (human endometrial carcinoma), and HepG2 (human liver hepatocellular carcinoma) cells (Figure 7). CNs demonstrated no obvious toxicity to the four cancer cells under all the tested concentrations, indicating that Cs could be used as a safe drug carrier. However, MCNs could inhibit cell growth in a dose-dependant manner. In this experiment, we found that MIF had no effect at low concentration, and was apt to precipitate at high concentration because of its strong hydrophobicity leading to decreased activity, which was consistent with our previous report [33]. Compared with free MIF, MCNs showed enhanced anti-proliferative activity, indicating that the anticancer effects of MCNs could be due to the synergistic effects of Cs and MIF. Previous studies using other cancer cell lines and other anticancer drugs also found the anticancer effects of Cs and the synergistic interplay of CNs and anticancer drugs [35–36]. Besides, the sustained release of MIF from the CNs could be another reason for the enhanced anti-proliferative efficiency of MCN. In our previous report, we loaded MIF into mesoporous silica nanoparticles (MSNs) coated with aEpCAM (aE-MSN-M) to target circulating tumor cells for cancer metastasis prevention, and also found that MIF entrapped in aE-MSN-M increased its efficacy by sustained release to reduce drug crystallization [33]. These results suggested that MCNs might be a good drug delivery system for delivery of MIF for cancer therapy.

### Pharmacokinetic study

**Figure 8:**
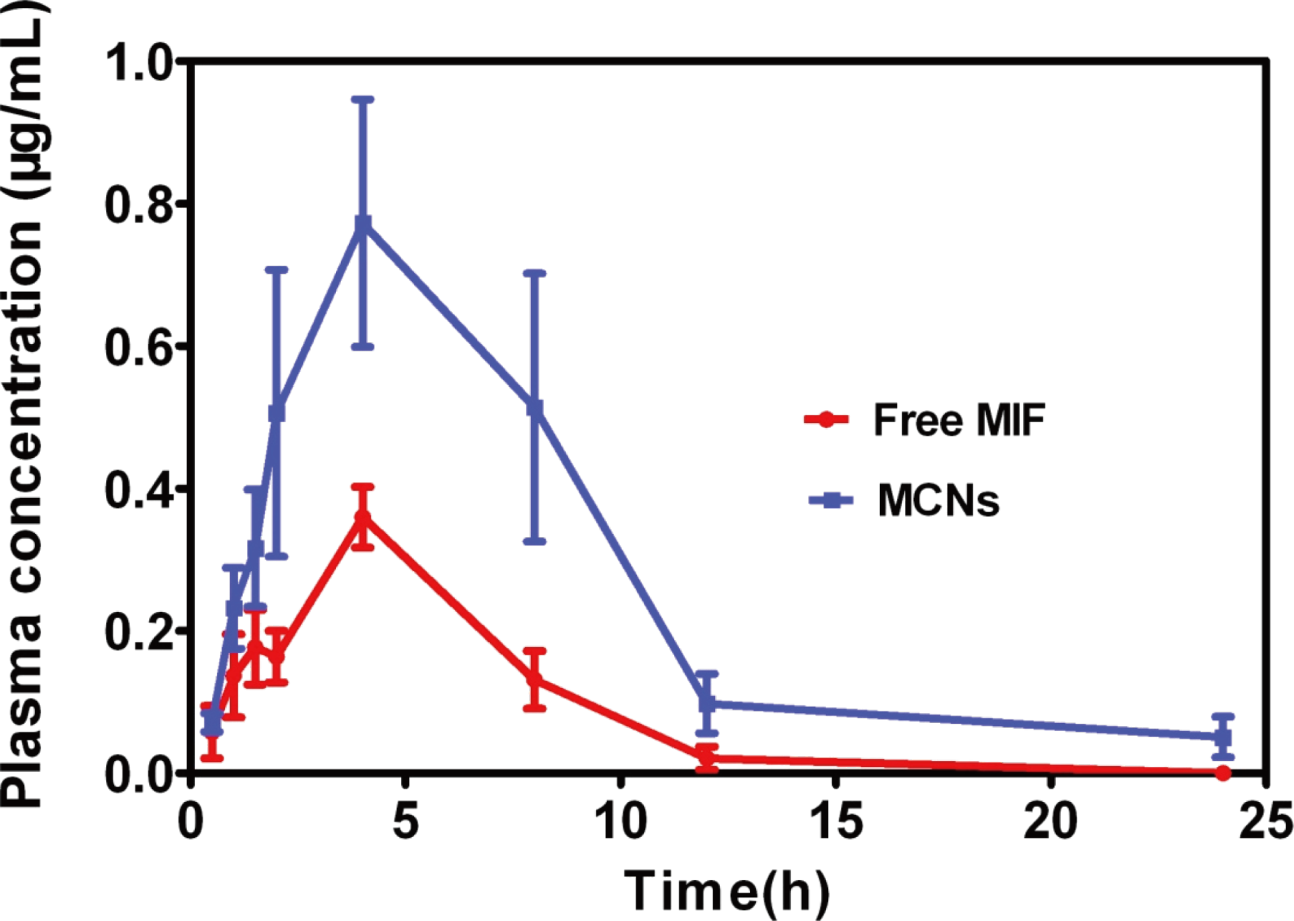
In vivo plasma concentration vs time of different MIF formulations. Male SD rats were given a single 30 mg/kg dose of MIF (in soybean oil solution) or a single dose of MCNs equivalent to the same dosage of MIF. Blood samples (each 0.5 mL) were collected into heparinized tubes from the orbital venous plexus at different time points after oral administration.

In our previous study, we found MIF showed distinct pharmacokinetic differences between genders. The bioavailability of MIF in male rats was significantly lower than in female rats [37]. Therefore, we chose male rats to perform the pharmacokinetic study to investigate whether MCNs could improve bioavailability of MIF. Following its oral administration, the plasma concentrations of MCNs were compared with pure MIF, and the mean plasma drug concentration–time curves were plotted (Figure 8). The corresponding pharmacokinetic parameters including elimination half-life (*t*_1/2_), area under the plasma concentration–time curve (AUC), the maximum plasma concentration (*C*_max_), and the time to maximum plasma concentration (*T*_max_) are presented in Table 1. The large error bars in the pharmacokinetics curve MCNs indicated that there are great individual differences in the disposition of MCNs. The statistical analysis indicated that significant differences in AUC_0−_*_t_* between MCNs and the MIF suspension. The AUC_0−∞_ value of the free MIF suspension and the MCNs were 2.4 ± 0.9 mg·h/mL and 6.8 ± 4.3 mg·h/mL, respectively. In addition, the *t*_1/2_ of the MCN group was longer than the MIF suspension group. These results demonstrated a relatively high and effective absorption of MCNs in vivo. The large surface area produced by the nanoparticles and the bioadhesive properties of Cs allow more of the drug to interact with the gastrointestinal tract [38–39]. These pharmacokinetic data clearly evidence the ability of CNs to enhance the absorption of MIF, which suggests that CNs could be a good formulation for MIF to increase its relative bioavailability.

**Table 1:** Pharmacokinetic parameters of different MIF formulations.

Parameters	MIF suspension	MCNs

AUC_0−_*_t_* (mg/L·h)	2.0 ± 0.5	6.3 ± 3.8^a^
AUC_0−∞_ (mg/L·h)	2.4 ± 0.9	6.8 ± 4.3^b^
*t*_1/2_ (h)	3.0 ± 2.0	4.0 ± 2.8
*T*_max_ (h)	3.4 ± 1.2	5.0 ± 2.0
*C*_max_ (mg/L)	0.36 ± 0.09	0.79 ± 0.33**

^a^*p* < 0.05; ^b^*p* < 0.01 as compared with the MIF group by Student’s *t*-test.

## Conclusion

In conclusion, CNs were employed as a drug delivery system for MIF delivery to improve the bioavailability of MIF, and consequently, to enhance the antitumor effect of MIF by the auxiliary anticancer functionality of Cs. MCNs prepared by an ionic gelation method under optimum preparation conditions were spherical in shape with an average diameter of 200 nm and satisfied EE and DL. FTIR and XRD spectra verified that MIF was successfully encapsulated in CNs. MIF could be released from the CNs in a sustained-release manner. MCNs were shown to increase the anticancer activity of MIF in several cancer cell lines and improved the oral absorption of MIF in male rats. All these results suggest that the MCNs may be further developed as a potential delivery system for MIF for cancer therapy.

## Experimental

### Materials

Chitosan (deacetylation degree of 90% and MW = 60 kDa, Cs) was purchased from Boao Biotechnology Co., Ltd. (Shanghai, China). Sodium tripolyphosphate (TPP) was purchased from Aladdin Industrial Co., Ltd. (Shanghai, China). Tween-80 and ethanol were purchased from Sinopharm Chemical Reagent Co., Ltd. (Shanghai, China). The RPMI 1640 medium, Dulbeccos Modified Eagle Medium (DMEM), McCoy's 5A medium, antibiotics, and fetal bovine serum (FBS) were purchased from Life Technologies GmbH (Darmstadt, Germany). 3-(4,5-Dimethylthiazol-2-yl)-2,5-diphenyltetrazolium bromide (MTT) was obtained from Sigma-Aldrich. Mifepriston (MIF) with the purity > 98% was provided by Shanghai New Hualian Pharmaceutical Co., Ltd. (Shanghai, China). All other solvents and chemicals used were of analytical grade.

### Preparation of MCNs

Briefly, Cs was dissolved in 2% (w/v) acetic acid solution and the solution was left standing for 1 h before use. MIF was dissolved in mixed solution of anhydrous ethanol and Tween-80 (1:1 v/v) and added to the Cs solution at different Cs/MIF mass ratios. After fully degassing, the resulting mixture was added dropwise into agitated TPP solution (0.3% w/v) at different pH values and the suspension was stirred at room temperature for 30 min. After standing for 2 h, the resulting complex was filtered out, washed at least three times with deionized water to remove the residual TPP, and dried in a desiccator to keep the weight constant. The collected MCNs were ground and stored at 4 °C for further research. The blank CNs were prepared similarly without adding MIF.

### Characterization

Infrared spectra were analyzed using a FTIR spectrometer (Intelligent, Nicolet 360, USA) with KBr pellet. The samples were scanned from 500–4000 cm^−1^ (scan step of 4 cm^−1^).

X-ray powder diffraction patterns of MCNs were obtained by an XRD diffractometer (Miniflex II, Rigaku, Japan). The X-ray source was Cu Kα radiation (30 kV, 15 mA).

AFM images were obtained on a Multimode 8 AFM series (Bruker, USA) in tapping/AC mode. The mean particle size and zeta potential of the MCNs were determined by dynamic light scattering using a Nanotrac^®^ Wave Particle Size and Zeta Potential Analyzer (Microtrac Inc, Montgomeryville, PA).

### Encapsulation efficiency (EE) and drug loading (DL) capacity

The EE and the DL of MIF in the MCNs was analyzed by high performance liquid chromatography (HPLC). Briefly, an accurately weighed quantity of the MCN powder was suspended in a specified volume of anhydrous alcohol with vigorous stirring for a period of time. The supernatant was collected after centrifugation and the concentration of MIF was analyzed with a Waters-2695 HPLC system equipped with a 2487 UV detector. MIF was separated on an Inertstil ODS2 C18 (150 × 4.6 mm, 5 μm) column with the mobile phase of acetonitrile–water (80:20 v/v), injection volume of 20 μL, flow rate of 1.0 mL/min, ambient column temperature and detection wavelength of 302 nm [33].

The EE and DL of MCNs were analyzed using the following formulas:


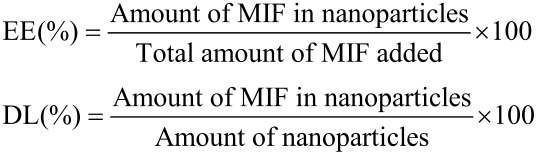


### In vitro drug release study

The in vitro release of MIF from the MCNs was performed using the dialysis bag diffusion technique [33]. The dialysis bag was cut into small pieces of appropriate length and boiled for 10 min in a volume (500 mL) of 2% (w/v) sodium bicarbonate and 1 mmol/L EDTA-2Na (pH 8.0) before use. Briefly, 10 mg of dried MCNs were resuspended in 10 mL of dissolution media; the suspension was then put into a pretreated dialysis bag which was sealed with a dialysis bag holder. The sealed dialysis bag was put into a large beaker containing 500 mL 1% ethanol/PBS solution of pH 7.4 or pH 2.5. The solution was stirred with a magnetic stirrer at 37 °C under a light-sealed condition. At certain time intervals, 1 mL of the release medium was taken out and the concentration of the released MIF was determined based on a free MIF calibration curve using the HPLC method as described above.

### In vitro anti-proliferative activity

#### Cell lines and cell culture

A549 human lung cancer cells, human epithelial carcinoma Hela cells, human endometrial carcinoma RL95-2 cells, and human hepatocellular liver carcinoma HepG2 cells were purchased from Type Culture Collection of the Chinese Academy of Sciences (Shanghai, China). A549 and Hela was grown in DMEM containing 10% FBS, 100 U/mL penicillin G sodium and 100 μg/mL streptomycin sulfate. RL95-2 and HepG2 cells were cultured in RMPI1640 mixed with 10% FBS (v/v), 100 U/mL penicillin G sodium, and 100 μg/mL streptomycin sulfate. The cells were incubated at 37 °C with 5% CO_2_ in a humid cell incubator.

#### MTT assay

A549, Hela, RL95-2 and HepG2 cells were seeded in a 96-well plate (8000 per well) and incubated for 24 h at 37 °C with 5% CO_2_. Blank CNs, free MIF, or MCNs were added to the well at predetermined drug concentrations (1, 10, 50, 100, 200 μg/mL), and incubated for 48 h at 37 °C with 5% CO_2_. The medium was removed and the cells were washed three times with PBS before incubation with MTT for 4 h at 37 °C. After the medium was removed, 100 μL of DMSO was added to the well for 20 min. The amount of MTT formazan product was analyzed spectro-photometrically at 570 nm by a TECAN Infinite F200 microplate reader.

### Pharmacokinetic study

#### Animals

Male Sprague-Dawley (SD) rats (180–220 g) were supplied by the Experimental Animal Center of Zhejiang Province and were housed with a 12 h dark/light cycle for three days before starting the experiment. The rats were fed a standard diet with water to drink ad libitum. Before drug administration, the rats were fasted overnight with free access to water. All studies involving animals were carried out in accordance with the National Nature Science Foundation of China (NSFC) regulation concerning the care and use of experimental animals and approved by our Animal Care and Use Committee to reduce the suffering and use of animals.

#### Oral administration

Eight male SD rats were randomly divided into two groups (*n* = 4). Group 1 was given a single 30 mg/kg dose of MIF (in soybean oil solution) and Group 2 was given a single dose of MCNs equivalent to the same dosage of MIF. Blood samples (each 0.5 mL) were collected in heparinized tubes from the orbital venous plexus at 0.5, 1, 1.5, 2, 4, 8, 12, and 24 h after oral administration. All blood samples were immediately processed by centrifugation at 4000 rpm for 8 min, and the plasma samples were stored at −20 °C before analysis.

#### Blood sample preparation

After the frozen plasma sample was thawed to room temperature, an aliquot of 200 μL of plasma was spiked with 50 μL levonorgestrel (internal standard, I.S., 98.0% purity) solution (426 ng/mL) in a 1.5 mL centrifuge tube and homogenized by vortex-mixing for 3 min. The mixed sample was then extracted with 2.0 mL of ethyl acetate by vortex-mixing for 3 min. After centrifugation at 4000 rpm for 10 min, the upper separated organic layer was carefully collected and evaporated to dryness under a gentle stream of nitrogen gas at 50 °C. The dried residue was reconstituted in 100 μL of methanol–water solution (50:50 v/v) followed by vortex-mixing for 3 min and then centrifuged at 15,000 rpm for 10 min. Afterwards, a 4 μL aliquot of the supernatant was injected into the chromatographic systems for analysis.

#### Quantification

The MIF concentration in plasma was determined using LC-MS/MS analysis according to the method reported earlier by Chen et al. [37] with slight modifications. Liquid chromatography was performed on an ACQUITY UPLC system using a BEH C_18_ column (50 mm × 2.1 mm, 1.7 μm, Waters Corporation, USA). The mobile phase solution was composed of methanol (A) and aqueous 0.1% (v/v) formic acid (B) with a gradient program as follows: 0–1.0 min (40–95% A), 1.0–2.5 min (95–95% A), 2.5–2.8 min (95–40% A), 2.8–4.0 min (40–40% A). The column temperature and flow rate were 35 °C and 0.3 mL/min, respectively. The injection volume was 4 μL.

The mass spectrometer (Waters Corporation., Milford, MA, USA) was operated in positive mode and equipped with an electrospray ionization (ESI) source. The main operating parameters were optimized as follows: desolvation gas (nitrogen) 600 L/h, cone gas (nitrogen) 50 L/h, collision gas (argon) about 0.15 MPa, cone voltage 30 V, capillary voltage 3.2 kV, source temperature 110 °C, and desolvation temperature 350 °C. The detection was operated in the multiple reaction monitoring (MRM) mode, and the MRM transitions were *m*/*z* 430.2→134.0 for MIF, and *m*/*z* 313.3→109.0 for I.S., respectively.

MIF was found stable in plasma under the stability test conditions. The calibration curve exhibited good linearity in the range of 7.1–2840 ng/mL (*R*^2^ > 0.998). The calibration curves were fitted with a weighted (1/χ^2^) least-squares linear regression method. A typical regression equation for the calibration curve was *y* = 2.66736 χ + 0.110667, *R*^2^ = 0.9980. The average recovery of MIF from the isolated plasma solution was greater than 80%.

#### Pharmacokinetic parameters and statistics

Pharmacokinetic parameters were calculated by using DAS version 3.0 software (BioGuider Co., Shanghai, China) including elimination half-life (*t*_1/2_), and area under the plasma concentration–time curve (AUC). The maximum plasma concentration (*C*_max_) and the time to maximum plasma concentration (*T*_max_) were directly read from the experimental data. Statistical analyses of pharmacokinetic parameters were performed by ANOVA to analyze differences among groups with *p* < 0.05 as the level of significance.

### Pharmacokinetic study

Statistical analysis was performed using the Student’s *t*-test. The differences were considered significant for *p* < 0.05, and *p* < 0.01 was indicative of a very significant difference.
